# Nuts as a Part of Dietary Strategy to Improve Metabolic Biomarkers: A Narrative Review

**DOI:** 10.3389/fnut.2022.881843

**Published:** 2022-03-29

**Authors:** Leila Khalili, Thoraya Mohamed Elhassan A-Elgadir, Ayaz Khurram Mallick, Hesham Ali El Enshasy, R. Z. Sayyed

**Affiliations:** ^1^Department of Community Nutrition, Faculty of Nutrition and Food Sciences, Tabriz University of Medical Sciences, Tabriz, Iran; ^2^Department of Clinical Biochemistry, College of Medicine, King Khalid University, Abha, Saudi Arabia; ^3^Insitute of Bioproduct Development (IBD), Universiti Teknologi Malaysia (UTM), Skudai, Malaysia; ^4^School of Chemical and Energy Engineering, Faculty of Engineering, Universiti Teknologi Malaysia (UTM), Skudai, Malaysia; ^5^City of Scientific Research and Technology Applications (SRTA), Alexandria, Egypt; ^6^Department of Microbiology, PSGVP Mandal's Arts, Science, and Commerce College, Shahada, India

**Keywords:** lipid profile, oxidative stress, glycemic control (A1C), metabolic biomarkers, inflammation

## Abstract

**Background:**

Nuts are in the spotlight because of their association with improved health outcomes. We aimed to summarize the findings of previous studies to evaluate the impact of nuts consumption on glycaemic and lipid profile, inflammation, and oxidative stress.

**Methods:**

Electronic searches for observational and intervention studies were undertaken in PubMed, Embase, Web of Science, and Science Direct until 2022 for searching the studies aiming the application of different types of nuts and the beneficial effects of nuts in improving glycemia, dyslipidemia, inflammation, and oxidative stress.

**Results:**

Results from 56 interventional, 9 narrative and 3 systematic reviews, and 12 meta-analysis studies, aiming at the evaluating beneficial effects of different types of nuts on metabolic markers, showed that nut consumption could improve metabolic markers, including glycaemic factors, lipid profile, and inflammatory and oxidative stress parameters in both healthy and individuals with metabolic disorders in a type-, dose- and duration-dependent manner. According to their unique nutrient components, nuts can be known as a part of a healthy diet, resulting in improved metabolic biomarkers.

**Conclusion:**

Considering the efficacy of nuts in improving metabolic markers, incorporation of, incorporating nuts the effectiveness of nuts in improving metabolic markers, incorporating nuts in the diet may prevent the incidence or aggravation of chronic metabolic diseases. Considering the health benefits of the nuts' components, including essential micronutrients, if consumed in the appropriate dose and duration to provide the necessary amount of effective micronutrients to improve health, we will see an improvement in metabolic factors. At the same time, more research is required to determine the optimal type, dose, and duration of nut intervention with regards to metabolic control and reducing the risk of developing metabolic disorders.

## Introduction

Nuts are known as healthy foods in the Mediterranean diet (MeDiet) because of their unique nutritional contents, and their consumption has been recommended to populations worldwide (1). Tree nuts, such as cashew nuts, hazelnuts, Brazil nuts, walnuts, almonds, pistachios, macadamias, and peanuts, are nutrient-dense foods, each with a unique combination of nutrients. Generally, these foods contain healthy fatty acid profiles including monounsaturated (MUFA) and polyunsaturated (PUFA) fatty acids, soluble and insoluble fibers, protein, vitamin K, vitamin E, thiamine, folate, minerals such as copper, magnesium, selenium (Se), and potassium, and substances such as antioxidants, phytosterols compounds, and xanthophyll carotenoids, with known health benefits for humans (2, 3). Due to their low levels of saturated fatty acids and high levels of unsaturated fatty acids and bioactive compounds, nuts have been reported to decrease the risk of cardio-metabolic disorders (4). The beneficial effects of nuts consumption on chronic disorders have been studied in previous research. Prior trials have recommended that regular nut consumption can provide beneficial effects on health outcomes and cardio-metabolic disorders, such as hypertension (5), cardiovascular diseases (2), obesity (6), and diabetes mellitus (7), with a reduction in chronic diseases mediators such as inflammation, oxidative stress, hyper-glycemia, visceral adiposity, endothelial dysfunction, and insulin resistance (8). Moreover, several meta-analyses of clinical trials and observational studies support the beneficial effects of nuts consumption on several cardio-metabolic disorders (9–13).

Nuts consumption offers a wide range of health benefits on humans; however, the present review will provide an update for giving an overview of recent findings of focus on metabolic benefits of nuts on glycaemic control, lipid profile, oxidative stress, and inflammatory status, and the appropriate dose and duration of nuts consumption to achieve metabolic benefits.

## Nuts and Metabolic Biomarkers

**Figure 1:**
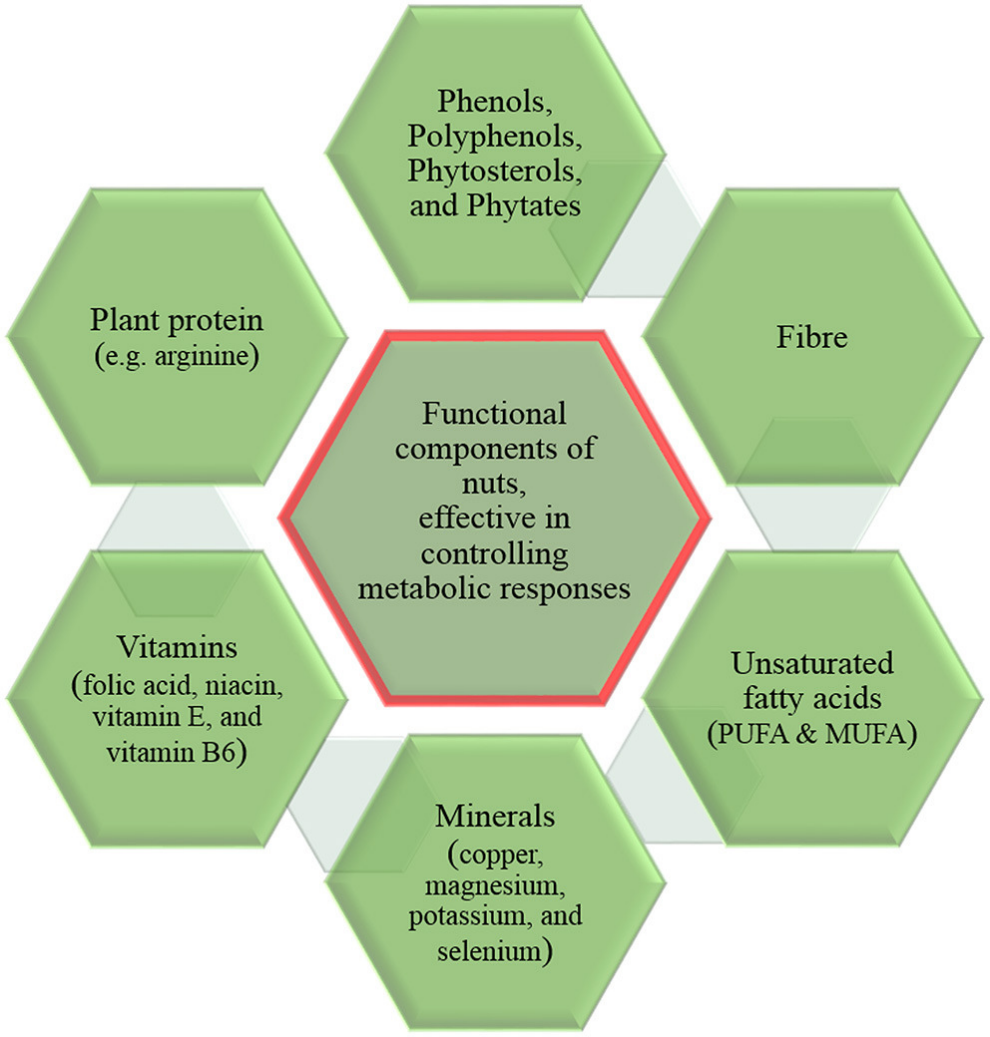
Beneficial components of nuts offering health benefits.

Evidence of epidemiological and interventional studies suggest that nuts consumption is associated with in reducing the incidence and aggravation of some metabolic diseases (14). Nuts are good sources of fiber, healthy fats, and other beneficial nutrients (Figure 1) (15), and each type of nut offers unique nutritional benefits.

**Figure 2:**
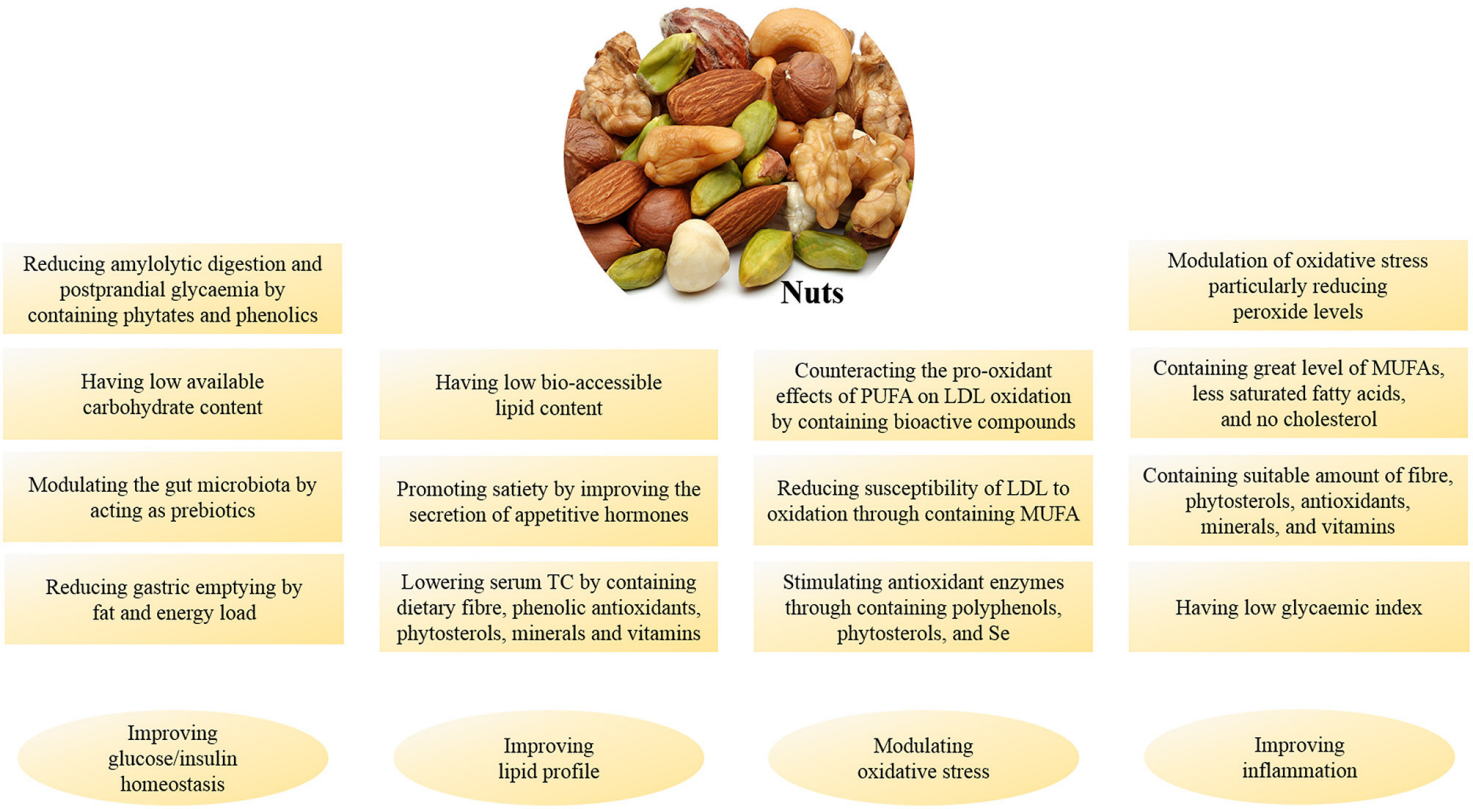
A summary of nuts mechanisms of action in controlling metabolic responses.

The ability of nuts to reduce the risk of chronic diseases and improving the level of metabolic markers is now well recognized (Figure 2) (16). Previous studies have shown that nuts consumption is associated with a reduced risk of cardiovascular disease (CVD), diabetes, and other chronic metabolic disorders (Table 1).

**Table 1:** A summary of studies evaluating the effect of nuts supplementation on metabolic markers.

**N**	**ID**	**Type of study**	**Study population**	**Dose**	**Duration**	**Findings**
**Almond**						
1	Li et al. (17)	Randomized crossover clinical trial	Chinese patients with T2DM	60 g/day	4 weeks	Those in the almond diet had lower levels of fasting insulin, FBS, and HOMA.IR.
2	Abazarfard et al. (18)	Randomized controlled trial (RCT)	Overweight and obese women	50 g/day	12 weeks	TC, TG, LDL-C, and FBS decreased significantly in the almond group compared to the nut-free group.
3	Ashley et al. (19)	Randomized crossover trial	Healthy individuals and individuals with T2DM	One serving per meal	12 weeks	A standard serving of almonds reduced postprandial glycaemia significantly in participants with diabetes but did not influence glycaemia in participants without diabetes.
4	Jung et al. (20)	Randomized, crossover trial	Overweight and obese Korean adults	56 g/day	4 weeks	Almond consumption decreased TC, LDL-C, and non-HDL-C, compared to the control. Of serum inflammatory markers, IL-10 was decreased by almond intake, and IL-1β and IL-6 tended to be lower with almonds, compared to the control.
5	Gulati et al. (21)	Free-living pre-post intervention study	Asian Indians in North India with T2DM	20% of energy intake	24 weeks	TC, TG, LDL-C, HbA1c, and hs-CRP significant improved after intervention.
6	Chen et al. (22)	RCT	Patients with T2DM		12 weeks	Almond decreased post-interventional FBS and HbA1c as compared to that of control.
7	Foster et al. (23)	RCT	Overweight and obese individuals	24 almonds per day	24 weeks	The almond-enriched diet, compared with the hypo-caloric nut-free diet, was associated with greater reductions in TC, total:HDL-C, and TG.
8	Liu et al. (24)	RCT	Healthy adults	56 g/day	20 weeks	Participants in the almond group showed favorable significant changes in including levels of TG, TC, LDL-C, and non-HDL-C after consuming of almond compared with those at baseline.
9	Sabaté et al. (25)	Randomized crossover design	Healthy subjects	10%, and 20% of total energy	4 weeks	Compared with the Step I diet, the high-almond diet reduced TC, LDL-C, Apo B, and ratio of LDL to HDL-C, and increased HDL-C.
10	Berryman et al. (26)	Randomized, crossover, controlled-feeding study	Individuals with elevated LDL-C	1.5 oz./day	6 weeks	The almond diet, compared with the control diet, decreased non-HDL-C and LDL-C
11	Liu et al. (27)	RCT	Young Korean adults	56 g/day	16 weeks	Consuming almonds as a daily snack reduced the levels of TC and LDL-C.
12	Liu et al. (28)	Randomized crossover controlled feeding trial	Chinese patients with T2DM	56 g/day	4 weeks	Compared to the control diet, the almond diet decreased IL-6, CRP, and TNF-α. The almond diet also enhanced the resistance of LDL against Cu^2+^-induced oxidation compared to the control diet.
13	Jia et al. (29)	Pilot study	Healthy adult male regular smokers	84 g/day	4 weeks	MDA levels in the almond-treated groups were lower than the controls. Almond consumption has preventive effects on oxidative stress caused by smoking.
14	Li et al. (30)	Randomized, crossover clinical trial	Healthy smoker male soldiers	84 g/day	4 weeks	After the almond intervention, serum α-tocopherol, SOD, and GPX increased and MDA decreased significantly in smokers.
15	Sweazea et al. (31)	Randomized, parallel-arm controlled study	Individuals with T2DM	1.5 oz/day	12 weeks	The inflammatory biomarker CRP was significantly reduced in the almond-treated group vs. controls.
**Pistachios**						
16	Sauder et al. (32)	Randomized, crossover, controlled feeding study	Adults with well-controlled T2DM	20% of total energy intake	4 weeks	TC, the ratio of total to HDL-C, and TG were significantly lower following the pistachio diet compared to the control diet.
17	Hern'andez-Alonso et al. (33)	RCT	Prediabetic subjects	57 g/day	16 weeks	FBS, insulin, and HOMA.IR decreased significantly after the intervention period compared with the control group.
18	Parham et al. (34)	double-blind, randomized, placebo-controlled, crossover trial	Patients with T2DM	50g/day	12 weeks	There was a marked decrease in HbA1c, FBS, and CRP in the pistachio group compared with the control group.
19	Gulati et al. (35)	RCT	Individuals with the MetS	20% energy	24 weeks	FBG, TC, LDL-C, hs-CRP, TNF-α, TBARS, and adiponectin levels were significant improved in the intervention group compared with control group.
20	Sari et al. (36)	RCT	Healthy young men	20% of daily caloric intake	4 weeks	Compared with the MeDiet, the pistachio diet decreased FBS, LDL-C, TC, TC/HDL-C and LDL-C/HDL-C ratios, and TG significantly. The pistachio diet significantly decreased serum IL-6, total oxidant status, lipid hydroperoxide, and MDA and increased SOD.
21	Canudas et al. (37)	Randomized crossover clinical trial	Prediabetic subjects	57 g/day	16 weeks	Compared with the control diet, the pistachio diet reduced oxidative damage to DNA and improved FBS and HOMA.IR.
**Walnut**						
22	Wu et al. (38)	Randomized, controlled, cross-over study	Healthy Caucasian men and post-menopausal women	43 g/day	8 weeks	Walnut supplementation significantly decreased fasting non-HDL-C and Apo B in healthy senior individuals.
23	Hwang et al. (39)	Two-arm, randomized, controlled crossover study	Korean adults with MetS	45 g/day	16 weeks	Significant improvements after walnut intake, compared to control intervention, in HDL-C, FBS, and HbA1c were observed.
24	Ros et al. (40)	Randomized crossover trial	Hypercholesterolemic men and women	32% of the energy from MUFA	4 weeks	The walnut diet significantly reduced TC and LDL-C.
25	Ashraf et al. (41)	Experimental study	Individuals with hyperlipidemia	25 g and 50 g/day	8 weeks	Consumption of walnut showed significant improvements in lipid profile of hyperlipidemic individuals.
26	Zambo'n et al. (42)	Randomized, crossover feeding trial	Men and women with polygenic hypercholesterolemia	35% of the energy obtained from MUFA	6 weeks	Compared with the MeDiet, the walnut diet produced significant changes in level of TC, LDL-C, and lipoprotein (a).
27	Bashan et al. (43)	RCT	Patients with dyslipidemia	40–50 g/day	12 weeks	TC, LDL-C, VLDL-C, and TG levels significantly decreased and HDL-C levels significantly increased in the walnut group at the end of the trial.
28	Torabian et al. (44)	Randomized crossover trial	Subjects with normal to moderate high plasma total cholesterol	12% of total daily energy intake	24 weeks	Significant changes in serum concentrations of TC and TG were seen and nearly significant changes in LDL-C were found by supplementing a habitual diet with walnuts.
29	Bamberger et al. (45)	Randomized, controlled, prospective, cross-over study	Healthy subjects	43 g/day	8 weeks	The walnut diet resulted in a significant reduction in fasting cholesterol, non-HDL-C, LDL-C, TG, and Apo B levels.
30	Rock et al. (46)	RCT	Overweight and obese men and women	15% of energy	24 weeks	The walnut-enriched diet group reduced TC and LDL-C.
31	Alibabaie et al. (47)	RCT	Female Undergraduate Students	40 g/day	4 weeks	A significant reduction was observed in the serum levels of LDL-C and TG after the consumption of walnuts.
**Peanuts**						
32	Liu et al. (48)	Randomized, controlled, crossover postprandial study	Healthy overweight or obese men	85 g		Acute peanut consumption blunted the serum TG.
**Hazelnuts**						
33	Damavandi et al. (49)	Controlled randomized parallel study	Patients with type 2 Diabetes	29 g/day	8 weeks	Hazelnut consumption non-significantly reduced TG, FBS, TC, and LDL-C levels.
34	Renzo et al. (50)	Prospective pilot clinical trial	Healthy volunteers	40 g/day	6 weeks	Significant up-regulation was detected for SOD, CAT, PPAR-γ, and ACE at the end of the study.
**Brazilian nut**						
35	Cominetti et al. (51)	RCT	Obese women	One nut per day	8 weeks	Obese people who implement daily consumption of Brazilian nuts could improve lipid profile, especially HDL-C levels
36	Colpo et al. (52)	Randomized crossover study	Healthy individuals	20 or 50 g		A single intake of Brazil nuts caused a significant decrease in serum IL-1, IL-6, TNF-α, and IFN-γ levels.
37	Maranhão et al. (53)	RCT	Obese female adolescents	15–25 g/day	16 weeks	Compared to placebo group, Brazil nuts intake reduced TC, TG, and LDL-ox
38	Macan et al. (54)	RCT	Patients with T2DM	One nut per day	24 weeks	Supplementation with Brazil nuts significantly increased serum Se levels. Furthermore, it was found that the cells were more resistant to H_2_O_2_-induced DNA damage after the supplementation.
39	Stockler-Pinto et al. (55)	RCT	Hemodialysis patients	One nut per day	12 weeks	The plasma Se and GPx activity increased; moreover, HDL-C levels increased and LDL-C levels decreased significantly after supplementation.
40	Watanabe et al. (56)	RCT	Patients in regular use of statins	One nut per day	12 weeks	Brazil nut decreased levels of CK activity in serum, MDA, and SOD and increased levels of GPX activity. Moreover, the supplementation caused significantly positive changes in plasma and erythrocyte Se concentrations.
**Cashew**						
41	Mah et al. (57)	Randomized, crossover, isocaloric, controlled-feeding study	Normally active men and women	28-64 g/day (11% of total energy intake)	4 weeks	Consumption of the cashew diet resulted in a significant change from baseline (compared with the control) in TC, LDL-C, non-HDL-C, and the TC:HDL-C ratio.
42	Damavandi et al. (58)	Randomized, isocaloric, controlled-feeding study	Patients with T2DM	10% of total calorie intake	8 weeks	Serum insulin, HOMA-IR, and LDL-C/HDL-C ratio significantly decreased in the cashew group compared with those of the controls.
43	Shidfar et al. (59)	Randomized parallel clinical trial	Patients with T2DM	10% of total daily calorie intake	8 weeks	Mean HDL-C and insulin concentration were significantly improved in intervention group compared with control group.
44	Mohan et al. (60)	Parallel-arm, randomized controlled trial	Patients with T2DM	30 g/day	12 weeks	Participants in the intervention group had a greater increase in plasma HDL-C compared with controls.
**Pecan**						
45	McKay et al. (61)	Randomized, controlled feeding trial	Patients with T2DM	15% of total calories	4 weeks	Changes in serum insulin, HOMA-IR, and HOMA-β were significantly greater in intervention group than those in control group.
46	Campos et al. (62)	RCT	Patients with stable coronary artery disease	30 g/day	12 weeks	The pecan nut consumption exhibited a significant reduction in non-HDL-C levels and in the TC/HDL-C ratio compared to the control group.
**Soy nut**						
47	Sedaghat et al. (63)	Case-control study	Patients with T2DM	60 g/day	8 weeks	Soy consumption significantly lowered FPG, HbA1c, plasma insulin levels, insulin-resistance, TC, and LDL-C.
48	Sedaghat et al. (64)	RCT	Patients with T2DM	60 g/day	8 weeks	Consuming soy nut significantly decreased the FBS, TC, and LDL-C and increased the capacity of serum total antioxidants.
49	Bakhtiary et al. (65)	RCT	Women with MetS	35 g/day	12 weeks	The soy-nut improved FBG, insulin, HOMA-IR, MDA, and TAC significantly after intervention.
50	Bakhtiari et al. (66)	RCT	Old women with MetS	35 g/day	12 weeks	Soy-nut significantly decreased TC, LDL-C, VLDL-C, Apo B100, FBS, serum insulin, HOMA-IR, and MDA levels. Moreover, the intervention significantly increased TAC compared with the control group.
51	Karamali et al. (67)	RCT	Women with polycystic ovary syndrome	35% daily protein intake	8 weeks	Consumption of soy-nut, compared with the control group, resulted in significant decreases in FBS, insulin, and insulin resistance, as well as a significant increase in quantitative insulin sensitivity check index. In addition, significant decreases in TC, TG, and MDA and significant increases in NO and GSH were seen in the test group compared to the control.
52	Azadbakht et al. (68)	Randomized crossover clinical trial	Postmenopausal women with the MetS	30 g/day	8 weeks	The soy-nut regimen significantly decreased HOMA.IR, FBS, and LDL-C compared with the soy-protein or control.
53	Hematdar et al. (69)	RCT	Subjects with T2DM	a cup of cooked soy beans three days a week	8 weeks	A significant decrease was observed in serum CRP of soy bean group which was significantly more than the controls.
**Baru almond**						
54	Bento et al. (70)	Randomized, crossover, placebo-controlled study	Mildly hypercholesterolemic subjects	20 g/day	6 weeks	Compared to placebo, supplementation of baru almonds reduced TC, LDL-C, and non-HDL-C.
55	Souza et al. (71)	RCT	Overweight and obese women	20 g/day	8 weeks	The consumption of baru almonds increased HDL-C level compared to baru almond-free diet.
56	Souza et al. (72)	Parallel-arm, randomized placebo-controlled trial	Overweight and obese women	20 g/day	8 weeks	The baru almond group increased the activity of GPx and plasma copper concentration when compared to the placebo group.

*FBS, fasting blood sugar; HOMA.IR, homeostatic model assessment for insulin resistance; HOMA-β, homeostasis model assessment of β-cell function; HbA1c, glycated hemoglobin; TC, total cholesterol; TG, triglyceride; LDL-C, low-density lipoprotein cholesterol; HDL-C, high-density lipoprotein cholesterol; IL, interleukin; hs-CRP, high-sensitivity C-reactive protein; TNF-α, tumor necrosis factor alpha; MDA, Malondialdehyde; TAC, total antioxidan capacity; SOD, superoxide dismutase; GPX, glutathione peroxidase; TBARS, thiobarbituric acid reactive substances; CAT, catalase; ACE, angiotensin-converting enzyme; PPAR-γ, peroxisome-proliferator activator receptor γ; FPG, form-amido-pyrimidine glycosylase; NO, nitric oxide; Apo B, apolipoprotein B; T2DM, type 2 diabetes mellitus; MetS, metabolic syndrome*.

### Glycaemic Control

A significant number of studies showed a link between regular nuts consumption and a reduction in risk of heart and metabolic disorders (73–76). Moreover, studies have shown that nuts consumption can improve glycemic responses in healthy and diabetic individuals (73). Nuts affect glycaemic response in a dose-dependent manner (77). According to the previous investigations, nuts help decrease glycaemic excursions; moreover, consuming nuts with carbohydrate-rich foods could reduce the postprandial impact on the insulin demand (77–80). The dose and duration of supplementation in studies that observed a significant effect of nuts consumption on glycaemic factors ranged from 30–60 g/day and 4–24 weeks, respectively (17, 18, 33, 35, 49, 52). The dose-dependent improvement in the glycaemic response to the meal has been revealed in previous investigations. In a study conducted on 10 healthy volunteers, it has been shown that the addition of 28 g of pistachios to white bread could improve glycaemic response, and this improvement was greater with the addition of 84 g of pistachios (79). In another study conducted on normo-glycaemic and individuals with T2D, adding 30, 60, and 90 g nuts to white bread reduced the glycaemic response of the meal by 11.2 ± 11.6% (*P* = 0.354), 29.7 ± 12.2% (*P* = 0.031), and 53.5 ± 8.5% (*P* < 0.001) (80).

It has been shown that people who eat more nuts in their diet have been shown to have a higher cardioprotective profile of glucose/insulin homeostasis (81). Findings from previous studies indicate a significant reduction in fasting insulin levels or an improvement in insulin resistance following the nuts consumption in both healthy and diabetic individuals (82, 83). Results from the Nurses' Health Study cohort indicated that eating more than 5 servings/week of nuts reduces the risk of diabetes compared to rare or no consumption (RR = 0.73, 95% CI = 0.60–0.89; P < .001) (84). Beneficial evidence is also supported by randomized controlled trials (RCTs). A meta-analysis of 12 RCTs with more than a 3-week follow-up period showed that consumption of a median dose of 56 g/day tree nuts could improve glycemic control in individuals with T2D compared to isocaloric diet without tree nuts (7). Additionally, the results of the study, conducted by Kendall et al. (80), showed that nuts eaten alone or with a high glycaemic index (GI) diet can lower postprandial blood sugar (PBG).

In addition to fat and protein, nuts have been reported to be rich sources of phenolics and phytates, both of which can decrease amylolytic digestion and postprandial blood sugar (85). In nuts, abundant fiber and polyphenols (flavonoids and non-flavonoids) may have a prebiotic effect and affect glucose metabolism (86). Several researchers have revealed that prebiotics' modulation of gut microbiota can improve glycemic control in healthy and diabetic subjects (87–89). Moreover, the blood sugar controlling effect of nuts can be attributed in part to the low carbohydrate content of nuts. Additionally, it has been shown that gastric emptying can be reduced by energy and fat load. Therefore, increasing the energy and fat load by increasing the dose of consumed nuts can partly explain the dose-dependent decrease in blood sugar in response to nuts consumption (80). In addition, it has also been shown that nuts can reduce the glycaemic response to a meal compared to a balanced meal in terms of energy and macronutrient contents (78). This can be due to nutsnuts' high unsaturated fat content, unsaturated fat content,and their unique physical structure. Unsaturated fatty acids in nuts in place of saturated fat and carbohydrate appear to, improve fasting, improve fasting and 2-h glucose levels significantly, but further studies should be performed with careful consideration of different types and amounts of nuts (90, 91).

In summary, overall nut intake has been revealed to be inversely associated with glycaemic factors. It may delay the development and progression of chronic metabolic diseases related to impaired glucose tolerance or glycaemic response.

### Lipid Profile

The beneficial effects of nuts consumption on lipid profiles, especially total cholesterol (TC) and low-density lipoprotein cholesterol (LDL-C), have been reported in clinical studies in both healthy and high-cholesterolemic individuals from various geographical areas (92). However, the effects varied based on the type and amount of nut, consumption duration, characteristics of the studied individuals, and study design (93–98). Most studies in this field that have shown a significant effect of nuts consumption on lipid factors have reported a dose of 20–64 g/day and a duration of 4–24 weeks (18, 20, 21, 53, 57, 70).

A Systematic Review conducted by Altamimi et al. (99) evaluated several dietary intervention studies that examined the effect of eating nuts on blood lipid levels. Analyses were performed on different types of nuts. Most of the studies showed improvement in lipid profile, including TC, LDL-C, high-density lipoprotein cholesterol (HDL-C), triglycerides (TG), and total cholesterol/high-density lipoprotein cholesterol (TC/HDL-C) after nut consumption. Tapsell et al. (95) found that following a healthy diet enriched with 30 g of walnuts for 6 months caused a significant reduction in LDL-C and increased HDL-C in subjects with diabetes. A recent study also showed beneficial changes in lipid profile after a MeDiet enriched with 30 g of mixed nuts compared to the control diet in high-risk CVD patients, about half of whom with T2D (96). Compared to the low-fat diet, the average changes in the MeDiet enriched with the nuts group were −6.20 (*P* = 0.040) for TC level, −13.0 (*p* = 0.022) for TG level, and −0.26 (*P* = 0.002) for the TC/HDL-C ratio (96). Lee et al. (97) showed that supplementing 30 g/day of mixed nuts (walnuts, peanuts, and pine nuts) with a usual diet for 6 weeks had beneficial effects on lipid profile in women with MetS. Compared to those in the control group, TC and non-HDL-C levels significantly decreased in the nut group (*P* = 0.023 and *P* = 0.016, respectively). A cross-sectional study conducted to examine the relationship between nuts intake and lipid profile among Iranians showed that regular consumption of pistachios, almonds, hazelnuts, and walnuts is associated with a lower incidence of hyperlipidemia (98). Results of this study revealed that nuts consumption is significantly associated with decreased LDL-C, TG, and Apo B/Apo A in both men and women and decreased TC only in women. In a systematic review, Mukuddem-Petersen et al. (92) found a 2–16% reduction in TC and 2–19% in LDL-C among people who consumed nuts compared to those on controlled diets. In addition, a prospective cohort study with positive quality showed the same results of nuts consumption in reducing TC, non-HDL-C, LDL-C, and Apo B-100 concentrations in women with diabetes (100). Sabaté et al. (25) compared the effects of two different amounts of almonds with the effects of the National Cholesterol Education Program Step I on serum lipids and lipoproteins in healthy adults and those with mildly high cholesterol (Low and High almond diets for replaceing 10 and 20%, respectively, of energy of the Step I diet with almonds). They found that in addition to lowering LDL-C, the high-almond diet significantly reduced Apo B concentrations. Apo B is a component of serum LDL and VLDL and consequently reflects the concentration of atherogenic lipoprotein particles that confer risk of CVD (101). Sabaté et al. (25) observed a decreasing trend in Apo B concentration with increasing amounts of almonds in the diet, indicating a decrease in LDL-cholesterol concentration and the number of LDL particles.

Several claimed reasons explain the biological rationale of consuming nuts and improving the lipid profile. First, the bioavailability of fat content of nuts is low, which means that large amounts of this fat are excreted in the feces (102). Second, the crunchy texture of nuts promotes satiety because the mechanical action of chewing leads to the release of appetite suppressant hormones such as cholecystokinin, which ultimately leads to lower calorie and fat intake (103). Third, it is suggested that nut components other than fatty acids, such as vitamins (e.g., vitamin E, vitamin B6, niacin, and folic acid), minerals (e.g., magnesium, potassium, and copper), dietary fiber, plant protein (e.g., arginine), phytosterols, and phenolic antioxidants are also bioactive in lowering serum TC level (104). Recent evidence suggests that the phytosterols in nuts which are more hydrophobic than cholesterol, impair cholesterol absorption because their hydrocarbon molecule is larger and has a greater affinity for micelles than cholesterol (105). As a result, cholesterol is displaced from the micelles, and the amount available for absorption becomes more limited (106). Nuts are also a rich source of protein (nearly 25% of energy), especially high in L-arginine (107). L-arginine supplementation is usually recommended for people with dyslipidemia. It has been suggested that L-arginine ihelpmproves the lipid profile because of its potential in increasing nitric oxide (NO) production. Production of NO increases the activity of lipoprotein lipase and finally, improving the hydrolysis of TGs reduces its plasma concentration (108). A meta-analysis, conducted by Hadi et al. (109), concluded that L-arginine supplementation could significantly reduce blood TG levels; however, more studies are needed to prove its hypocholesterolemic effects.

Due to their unique nutrient profile, enriching a healthy diet with nuts may positively affecting lipid profile. Lipid-lowering effects of nuts should be considered in future food-based dietary strategies for improving plasma lipid levels.

### Inflammation

Inflammation plays a key role in the development of cardio-metabolic diseases, such as CVD and T2D and nuts may moderate inflammation and the development of endothelial dysfunction and cardio-metabolic disorders through their bioactive components such as L-arginine, alpha-linolenic acid (MUFA), polyphenols, and fiber (110). Cross-sectional studies in this field showed that people with regular nut consumption had lower serum concentrations of proinflammatory cytokines or endothelial cell adhesion molecules. Effective interventional periods ranged from 4 to 24 weeks, with doses ranging from 20 to 56 g/day (20, 21, 34, 52).

It has been shown that α-linolenic acid [18:3(n-3)], extracted from nut is inversely associated with interleukin-6 (IL-6), soluble tumor necrosis factor (TNF) receptors 1 and 2, fibrinogen, and C-reactive protein (CRP) levels in both healthy subjects and individuals with coronary artery disease (111, 112). Furthermore, subjects who ate more nuts showed lower levels of intercellular adhesion molecule 1 (ICAM-1)-1, vascular cell adhesion protein 1 (VCAM-1), CRP, and IL-6 (113). In a cross-sectional analysis of the Multi-Ethnic Study of Atherosclerosis, consumption of seed and nut was inversely associated with IL-6, CRP, and fibrinogen (114). Yu et al. examined the association of common consumption of nuts with inflammatory biomarkers in two large groups of American men and women (115). They showed that nuts consumption was inversely associated with the concentration of IL-6 and CRP. The health effects of nuts on inflammatory markers have also been investigated in clinical trials. In a randomized trial, patients with MetS were advised to follow a healthy diet with 30 g of daily supplementation of raw nuts for 12 weeks (7.5 g hazelnuts, 15 g walnuts, and 7.5 g almonds) (82). Among inflammatory markers, the diet of nuts significantly resulted in changes in plasma IL-6. In another clinical trial, almond diet (56 g/day) for 4 weeks reduced CRP by a median 10.3 % (95% CI: −24.1, 40.5), TNF-α by a median 15.7 % (95% CI: −0.3, 29.9), and IL-6 by a median 10.3 % (95% CI: 5.2, 12.6 %) in comparison with the control diet (28). Gulati et al. (35) showed that supplementation of pistachio (20% energy) for 24-wk significantly improved hs-CRP (*P* < 0.05) and TNF-α (*P* < 0.03) in individuals with MetS. A meta-analysis of 32 RCTs showed that consumption of nuts resulted in small and non-significant differences in CRP levels (10).

The beneficial effects of nuts on inflammatory markers are attributed to their composition, which is recognized by lesser saturated fatty acids, a greater level of mono-unsaturated fatty acids (MUFAs), no cholesterol, and a suitable amount of phytosterols, fiber, protein, antioxidants, and numerous vitamins and minerals (116, 117). An increasing number of studies have examined the effect of these nutrients on inflammation. As suggested in cross-sectional studies, the high content of phenolic compounds in nuts may anticipate the anti-inflammatory effect of regular consumption of nuts (100). Furthermore, nuts are rich sources of fiber and therefore have a low glycaemic index (GI). It has been reported that diets with low GI can reduce CRP levels (118). Nuts are an important source of antioxidants essential for human health (8). Modulation of oxidative stress (particularly reducing peroxide levels) can decrease inflammation. The fatty acid composition of nuts is beneficial because the content of saturated fatty acids (SFAs) is low (4–16%), and almost half of the total fat content is composed of unsaturated fats. In most nuts MUFA (oleic acid) and different amounts of PUFA (119). Earlier data indicate that MUFA and PUFAs can exhibit anti-inflammatory properties in various experimental models of inflammation (120). The mechanisms by which dietary fatty acids inhibit cytokine production are unknown but may be related to inhibition of the inflammatory cascade at the level of lipoxygenase (LOX) and cyclooxygenase (COX) (121). Regarding the protective effect of nuts, it has been reported that the MeDiet enriched with nuts can lower the incidence of major cardiovascular events among people at high risk of cardiovascular diseases (122).

Considering existing evidence regular consumption of nuts can be associated with a nutritional profile of inflammatory biomarkers. However, further studies are needed to definitively address the important question of the anti-inflammatory effects of nuts.

### Oxidative Stress

Consumption of nuts as food sources of antioxidants can prevent pro-oxidation and excessive oxidation of LDL (123). Consumption of antioxidant foods as components of the diet playss an important role in managing cardio-metabolic disorders (124). Vitamin E and phenolic compounds are among the nutrients with antioxidant activities that nuts are a rich source of both. (125). *In vitro* studies (126, 127), animal models (128, 129), observational studies (130), and randomized trials (35, 72) suggest the potential benefits of including various nuts in the diet with regards to oxidative stress biomarkers. Most of the studies that have reported the potential beneficial effects of eating nuts on oxidative stress have focused on pistachios, almonds, peanuts, or walnuts, all of which are rich sources of MUFA. For instance, a sub-analysis of the PREDIMED (Prevención con Dieta Mediterránea) study, which assessed biomarkers associated with oxidative stress, identified higher superoxide dismutase (SOD) and catalase activity and lower plasma xanthine oxidase activity in the intervention group (the MeDiet + extra-virgin olive oil and the MeDiet + nuts) (131). The effective dose and duration observed in clinical studies were 20–84 g/day and 4–16 weeks, respectively (28, 30, 50, 53, 65, 72).

The nutritional intervention of 1 unit/d (20 g/day) of Brazil nuts for 3 months in hemodialysis patients resulted in improved plasma glutathione peroxidase (GPx) levels and a reduction in 8-isoprostane and 8-hydroxy-2′-deoxyguanosine (8-OHdG) levels (*p* < 0.001) (55). A clinical trial on 46 overweight and obese women found that after 8 weeks of intervention, the Baru nuts group (20 g/day) showed a significant increase in GPx activity compared to the placebo group (72). Jenkins et al. (132) showed that a full dose of almond supplement (73 ± 3 g/day, 22% of energy) or half a dose over 4 weeks reduced oxidized LDL in subjects with dyslipidemia. Liu et al. (28) found similar results after 4 weeks of supplementation with almond (~ 6 g/day) in individuals with dyslipidemia and T2D. Pistachio supplementation (10 or 20% of energy) for 4 weeks was also effective in reducing oxidized LDL in people with hyperlipidemia (133).

It has been revealed that bioactive compounds (phytosterols, polyphenols), MUFA, Se, and tocopherols are the main factors in the beneficial effects of nuts in modulating oxidative stress. These agents can also reduce the pro-oxidant effects of PUFA on LDL oxidation and DNA damages (134). The main fat compounds in hazelnuts and almonds are MUFA and are associated with decreased LDL sensitivity to oxidation (119). Therefore, differences in the fat content of different nuts may partly explain why pistachios, walnuts, almonds, and other MUFA-rich nuts can reduce oxidative stress, whereas walnuts (PUFA-rich) Do not have. Se, phytosterols, and polyphenols can activate the Nrf2 (nuclear factor erythroid 2-related factor 2) pathway (134). Nrf2 stimulates the antioxidant response element (ARE) genes transcription and encodes the antioxidant (e.g., GPx) and detoxifying enzymes (55). The Nfr2 pathway also activates NQO1, stabilizing proteins and protecting them against oxidative degradation (135).

Consumption of antioxidant food sources and/or antioxidant supplements seems to contribute to the prevention and/or treatment of metabolic disorders through widely known mechanisms; nuts are a good example of such food sources because of their good taste and dose-effect that allows them to be included in diets.

Based on the findings of evaluated studies, depending on the dose and duration of consumption (Table 2), mixed nuts can have health effects on metabolic markers, including glycaemic response, lipid factors, oxidative stress, and inflammation. In contrast, no adverse effects of nuts intake on metabolic biomarkers have been found in clinical trials (136).

**Table 2:** Appropriate dose and duration of mixed nut consumption to insert metabolic efficacy.

**Metabolic response**	**Optimal dose (g/day)**	**Optimal duration (weeks)**
Glycaemic response	28–60	4–24
Lipid profile	20–64	4–24
Inflammatory response	20–56	4–24
Oxidative stress markers	20–84	4–16

## Conclusions

As ready-to-eat snack foods, nuts have healthy lipid components and are excellent sources of fiber and some micronutrients. The US FDA has approved a qualified health claim for nuts suggesting that daily consumption of nuts may help to reduce the risk of some chronic disorders purportedly through the improvement of metabolic markers. However, the intervention's selected type, dose, and duration should be based on the therapeutic goals. For example, the effectiveness of different types of nuts including, Almond, Pistachios, Walnut, Hazelnuts, Brazilian nut, Peanuts, Cashew, Pecan, and Soy nut on glycaemic response and lipid factors has been shown in several clinical trials. But, in the case of inflammation, the effectiveness of walnuts has been more supported due to their high ALA and phenolic compounds. Additionally, in the case of oxidative stress, the effectiveness of almonds, pistachios, pecans, or peanuts due to their rich sources of MUFA has been supported. Further research is needed to establish the intakes of different varieties of nuts to deliver optimal metabolic benefits.

## Author Contributions

All authors conceived the idea, participated in the study design, drafted the manuscript, reviewed, edited, and approved the final manuscript.

## Funding

This work is funded by RMC-UTM with industrial Grant Nos. RJ130000.7609.4C187 and RJ130000.7609.4C284.

## Conflict of Interest

The authors declare that the research was conducted in the absence of any commercial or financial relationships that could be construed as a potential conflict of interest.

## Publisher's Note

All claims expressed in this article are solely those of the authors and do not necessarily represent those of their affiliated organizations, or those of the publisher, the editors and the reviewers. Any product that may be evaluated in this article, or claim that may be made by its manufacturer, is not guaranteed or endorsed by the publisher.
